# Mutual Modulation of the Activities of Human CYP2D6 and Four UGTs during the Metabolism of Propranolol

**DOI:** 10.3390/cimb45090451

**Published:** 2023-08-26

**Authors:** Fan Yang, Sangeeta Shrestha Sharma, Matthias Bureik, Maria Kristina Parr

**Affiliations:** 1 Pharmaceutical and Medicinal Chemistry (Pharmaceutical Analyses), Institute of Pharmacy, Freie Universität Berlin, 14195 Berlin, Germany; yangfano129@zedat.fu-berlin.de; 2 School of Pharmaceutical Science and Technology, Tianjin University, Tianjin 300072, China; sangee16@hotmail.com (S.S.S.); matthias@tju.edu.cn (M.B.)

**Keywords:** cytochrome P450s, UDP-glucuronosyltransferases, protein–protein interaction, drug metabolism

## Abstract

Cytochromes P450 (CYP) and UDP-glucuronosyltransferases (UGT) are two enzyme families that play an important role in drug metabolism, catalyzing either the functionalization or glucuronidation of xenobiotics. However, their mutual interactions are poorly understood. In this study, the functional interactions of human CYP2D6 with four human UGTs (UGT1A7, UGT1A8, UGT1A9, and UGT2A1) were investigated using our previously established co-expression model system in the fission yeast *Schizosaccharomyces pombe*. The substrate employed was propranolol because it is well metabolized by CYP2D6. Moreover, the CYP2D6 metabolite 4-hydroxypropranolol is a known substrate for the four UGTs included in this study. Co-expression of either UGT1A7, UGT1A8, or UGT1A9 was found to increase the activity of CYP2D6 by a factor of 3.3, 2.1 or 2.8, respectively, for the conversion of propranolol to 4-hydroxypropranolol. In contrast, UGT2A1 co-expression did not change CYP2D6 activity. On the other hand, the activities of all four UGTs were completely suppressed by co-expression of CYP2D6. This data corroborates our previous report that CYP2D6 is involved in functional CYP-UGT interactions and suggest that such interactions can contribute to both adverse drug reactions and changes in drug efficacy.

## 1. Introduction

Drug metabolism is the process in which xenobiotic compounds are converted to new compounds (metabolites). Active pharmaceutical ingredients (APIs) are often lipophilic and thus cannot be easily excreted from the body. They are, therefore, transformed by drug metabolizing enzymes (DMEs) into more hydrophilic metabolites [1]. Generally speaking, drug metabolism involves either the addition or unmasking of a polar group (phase I metabolism), and conjugation reactions (phase II metabolism) [2]. The cytochrome P450 (CYP) enzymes are a large superfamily of monooxygenases, with 57 CYPs reported in humans. Many of these catalyze the oxidation of drugs and other xenobiotics in phase I metabolism [3]. As CYPs can bind their substrate and oxygen, but not the cofactor NADPH, they depend on electron transfer proteins such as NADPH cytochrome P450 oxidoreductase (CPR or POR) for reduction [4]. Often, CYPs add hydroxy groups to lipophilic substrates, which may then undergo further conjugation reactions in phase II metabolization [5]. Uridine 5′-diphospho (UDP)-glucuronosyltransferases (UGTs) play a vital role in such phase II reactions. They catalyze the transfer of a glucuronic acid moiety from the cofactor UDP-glucuronic acid to a substrate which has a suited functional group, such as hydroxy or carboxy groups [6,7]. The human UGTs are able to conduct the conjugation reactions of a wide variety of drugs, environmental chemicals, and natural compounds, with the 19 members of UGT1 and UGT2 families appearing to be more important in drug metabolism than the other three enzymes [8].

Most mammalian CYPs are located on the cytoplasmic side of the endoplasmic reticulum, while the UGTs are embedded on the other side of the same membrane. Moreover, many members of both families are expressed in the same tissues [5]. Therefore, protein–protein interactions between these two types of enzymes are possible in vivo. Research in this field started about two decades ago with the observation that rat CYP1A1 was co-eluted with rat UGTs during affinity chromatography [9]. Subsequent studies showed that the glucuronidation of 3-hydroxy-benzo(a)pyrene by a rat UGT is hampered by a CYP1A inhibitor via a mechanism requiring intact microsomal membranes [10]. Immunoprecipitation experiments demonstrated the interaction of human CYP3A4 with human UGT1A1, UGT1A6, and UGT2B7 [11]. The results of these studies that identified candidates for CYP-UGT protein–protein binding were corroborated by other efforts directed at activity measurements: The effect of human CYP3A4 co-expression on human UGT2B7 was studied by monitoring the glucuronidation of morphine in CYP-UGT co-expressing COS-1 cells, and it was found that the K_m_ value was increased by almost tenfold compared to the UGT2B7 single-expressed samples [12]. Further studies indicated that the J-helix of CYP3A4 contributed to its interaction with UGT2B7 [13]. CYP3A4 was also reported to increase the activity of UGT1A1 and UGT1A7 for the glucuronidation of the irinotecan metabolite SN-38 [14]. On the other hand, the activity of CYP3A4 was found to be suppressed when UGT1A9 or UGT2B7 were co-expressed in a baculovirus–insect cell system [15,16].

Liver microsomes of human and rat were commonly used in the studies on the association of CYPs and UGTs with coimmunoprecipitation techniques [11]. However, liver microsomes may not be the ideal enzyme resource for investigating the functional interaction between CYPs and UGTs. Due to the presence of multiple human CYPs and UGTs in the liver, leading to a vast number of potential protein–protein interactions, data interpretation would be by far too complex. Furthermore, many substrates of both enzyme families are not specific to a single enzyme. Therefore, recombinant microbes that overexpress these enzymes offer a viable alternative for studying the functional interaction between CYPs and UGTs. The fission yeast *Schizosaccharomyces pombe* has been successfully utilized for expressing human CYPs and UGTs [17,18]. Recently, diploid fission yeast strains were developed, enabling the simultaneous expression of human CYPs (along with human CPR) and UGTs [19]. A previous study demonstrated evidence of a proper recognition of human subcellular localization sequences of human CYPs in fission yeast systems [20]. Furthermore, in a study where CYP1A1 and UGT1A6 were co-expressed in yeast, protease treatment of the microsomes showed the correct topological orientation of the UGT in the membranes [21]. This indicates that the topology of CYP and UGT enzymes expressed in yeasts should closely resemble that of the ER membrane in humans. In our previous study, co-expression experiments were conducted, involving each of the 19 human UGTs from families 1 and 2 with CYP2C9, CYP2D6, or CYP4Z1. A total of 72 interactions were monitored using proluciferin probe substrates. The results showed that the co-expression of UGTs had a significant effect on CYP activity in the majority of cases (88%), with both positive (58%) and negative (30%) effects observed. Conversely, the effect of CYP co-expression on UGT activity in the proluciferin study was generally less pronounced and primarily adverse.

**Figure 1:**
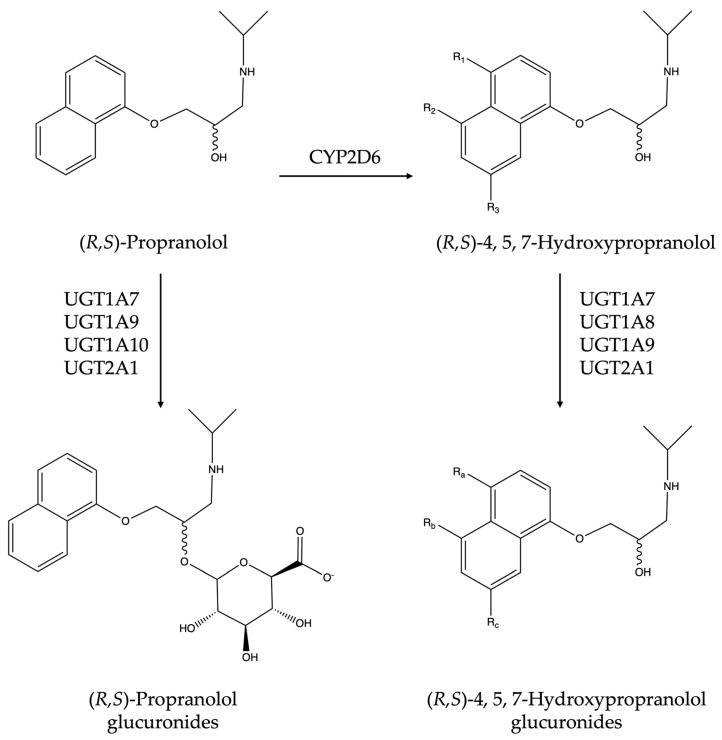
Possible metabolic pathways of propranolol in CYP2D6-UGTs co-expressing yeast cells. 4-Hydroxypropranolol: R_1_ = -OH, R_2_ = H, R_3_ = H; 5-hydroxypropranolol, R_1_ = H, R_2_ = -OH, R_3_ = H; 7-hydroxypropranolol: R_1_ = H, R_2_ = H, R_3_ = -OH; 4-hydroxypropranolol glucuronides: R_a_ = -O-glucuronic acid, R_b_ = H, R_c_ = H; 5-hydroxypropranolol glucuronides, R_a_ = H, R_b_ = -O-glucuronic acid, R_c_ = H; 7-hydroxypropranolol glucuronides: R_a_ = H, R_b_ = H, R_c_ = -O-glucuronic acid.

In the present study, this data is extended by moving on from proluciferin probe substrates to a drug compound that is known to be metabolized by both CYPs and UGTs. Propranolol is a beta-receptor blocker that is commonly used to treat various cardiovascular disorders [22,23,24,25]. This API has long been known to be metabolized in the human body mainly in three different pathways: (I) hydroxylation on the naphthalene ring at 4-, 5- and 7-positions; (II) glucuronidation of the aliphatic hydroxy group; and (III) *N*-desisopropylation on the side chain [26,27,28]. CYP2D6 mainly catalyzes the 4-, 5- and 7-hydroxy-lation of propranolol, and it also contributes to its *N*-desisopropylation [29]. However, the latter reaction is mainly catalyzed by CYP1A2, which also possesses a certain ability for ring hydroxylation [24]. In our previous study, propranolol was found to be glucuronidated by UGT1A7, UGT1A9, UGT1A10, and UGT2A1, while its metabolite 4-hydroxypropranolol was found to be converted by UGT1A7, UGT1A8, UGT1A9, and UGT2A1 [30]. The possible pathways of propranolol metabolism by CYP2D6 and these UGTs are illustrated in Figure 1.

In the present study, diploid fission yeast strains that co-express CYP2D6 with one of four UGTs each (UGT1A7, UGT1A8, UGT1A9, or UGT2A1) were chosen to investigate their mutual activity influences in the presence of propranolol. The changes in the hydroxy-lation activity of CYP2D6 and the glucuronidation activity of the UGTs were studied by monitoring the production of hydroxypropranolol metabolites and the respective glucuronides.

## 2. Materials and Methods

### 2.1. Chemicals and Reagents

Tris buffer, potassium chloride, glycerol and Triton X-100 were sourced from Carl Roth GmbH & Co. KG (Karlsruhe, Germany); UDPGA, (±)-propranolol, (*R*)-propranolol, and (±)-4-hydroxypropranolol were obtained from Sigma-Aldrich (Taufkirchen, Germany); (±)-5-hydroxypropranolol was purchased from Cayman Chemical Company (Ann Arbor, MI, USA); the NADPH regeneration system was sourced from Promega Corporation (Madison, WI, USA); and 4-methoxypropranolol was synthesized in-house as described previously [30,31,32]. Ammonium hydrogen carbonate was purchased from VWR International GmbH (Darmstadt, Germany); and phosphate buffered saline (PBS, pH 7.4) was purchased from VWR Chemicals LLC (Solon, OH, USA).

### 2.2. pH Extraction Assay

The extraction efficiency of propranolol, 4-hydroxypropranolol, and 4-methoxypropranolol by ethyl acetate from the reaction buffer was evaluated by applying different pH values. To a series of pH-adjusted solutions of 180 μL ammonium hydrogen carbonate buffer (50 mM, pH 5, 6, 7, 8, 9, 10, 11), 20 μL of compound stock solution (10 mM) was added, resulting in a final compound concentration of 1 mM. The mixture was vortex mixed for 1 min. Afterwards, 200 μL of ethyl acetate were added to the mixture. Then, the mixture was vortex mixed for 1 min and centrifuged for 1 min at 14,100× *g*. The organic phase was transferred to another 1.5 mL tube. Those extraction steps were repeated three times. The combined organic extracts were evaporated under N_2_ and the residue was redissolved in 200 μL of methanol and used for LC-MS/MS analysis. The aqueous residues were centrifuged at 14,100× *g* for 2 min to obtain supernatants and then used for LC-MS/MS analysis, as well. 

### 2.3. Metabolite Quantitation

A set of standard solutions of 4-hydroxypropranolol and 5-hydroxypropranolol was prepared by diluting stock solutions with methanol. The stock solutions of 4- and 5-hydroxypropranolol in methanol, at a concentration of 10 mM, were prepared from purchased standards and stored at 4 °C. The diluted concentrations ranged from 0.0001 μM to 1.0 μM (0.0001 μM, 0.0005 μM, 0.001 μM, 0.005 μM, 0.01 μM, 0.05 μM, 0.1 μM, 0.5 μM, 1 μM), with each concentration measured in triplicate using LC-MS/MS. The peak area of the analyte was plotted on the *Y*-axis, while the corresponding concentration was plotted on the *X*-axis to generate a calibration curve. The linearity of the curve was assessed by calculating the R^2^ value, which was found to be 0.99, indicating a strong linear relationship. The calibration curve was subsequently utilized to determine the concentrations of 4-hydroxypropranolol and 5-hydroxypropranolol, as presented in Section 3.2 of this article.

### 2.4. qPCR Analysis of CYP2D6 in Diploid Yeast Strains

Total RNA of the strains was isolated using the Eastep^®^ Super Total RNA Extraction Kit (Promega Biotech Co., Ltd., Beijing, China) according to the manufacturer’s instructions. The concentrations of isolated RNAs were determined using a TECAN Infinite^®^ F200 Pro microtiter plate reader (Thermo Fisher Scientific, Waltham, MA, USA). cDNA synthesis was performed with 800 ng of total RNA in 20 µL of reaction volume using the HiFiScript cDNA Synthesis Kit (CWBIO, Taizhou, China) according to the manufacturer’s instructions. The 20 µL of reaction volume was first incubated at 42 °C for 30 min and then at 85 °C for 5 min. The synthesized cDNA was then placed on ice for the subsequent qPCR analysis.

Quantitative expression of CYP2D6 was measured using two-step reverse transcription PCR. The primers used are listed in Table 1. In brief, 5 µL of cDNA and Ultra SYBR Mixture (Low ROX, CWBIO, Taizhou, China) in 25 µL reaction volume were amplified with the initial denaturation at 95 °C for 10 min followed by 35 thermal cycles of 98 °C for 10 s, 60 °C for 1 min, and 72 °C for 15 s. The Ct values of the target gene were normalized to the relative mean of the expression of the housekeeping gene *act1*. The fold change of the gene expression was determined by the 2^^−ΔΔCt^ method, as described [33]. The effect of four UGTs in CYP2D6 was determined by comparing the expression level with the control diploid strain expressing CYP2D6 and CPR (SAN300).

### 2.5. Yeast Strains, Preparation, and Long-Term Storage of Enzyme Bags

The construction of the diploid yeast strains was reported very recently [19]. All yeast strains used in this study are listed in Table 2. The preparation of permeabilized cells (enzyme bags) was conducted as described previously [34] but was changed with respect to the culturing time and the type of the medium for the diploid yeast. The Edinburgh Minimal Medium (EMM) was prepared in-house based on the published protocol [35]. Yeast strains SAN300, SAN306, SAN307, SAN308, and SAN310 were initially cultured on solid EMM supplemented with histidine at 30 °C for 5 days. Subsequently, the cells were transferred to 10 mL of liquid EMM containing histidine and incubated at 30 °C with agitation at 230 rpm for 24 h. Afterwards, 10 mL of the pre-cultures were transferred into 100 mL of liquid EMM with histidine in a 200 mL flask and incubated at 30 °C while agitating at 230 rpm for 48 h. The yeast cell density was counted under the microscope. 

The calculated volume of liquid medium containing 5 × 10^7^ yeast cells was used for a single-enzyme-bag reaction according to our previous study [30]. Following centrifugation at 3320× *g* for 5 min, the supernatant was discarded, and the cell pellets were obtained. To permeabilize the cell membrane, 1 mL of 0.3% Triton X-100 in Tris-KCL buffer was added to the tube, and the sample was incubated at 30 °C with agitation at 230 rpm for 1 h. After permeabilization, the cell pellets were washed three times with 1 mL of NH_4_HCO_3_ buffer (50 mM, pH 7.8) to remove the residual detergent. Subsequently, the cell pellets were resuspended in 200 μL of PBS containing 50% glycerol (*v*/*v*). Finally, the enzyme bag sample was flash frozen using liquid nitrogen and stored at −80 °C. Prior to substrate incubation, the frozen enzyme bags were thawed on ice and washed twice using reaction buffer to remove the glycerol. The stability of the enzymatic activity was monitored by analyzing metabolite production with freshly prepared enzyme bags as compared to enzyme bags stored for 1 day, 1 week, or 3 months. All enzyme bags used were prepared from the same batch and subjected to a single freeze–thaw cycle only.

### 2.6. Metabolization of Propranolol in Diploid Yeast Enzyme Bags

The frozen enzyme bags were thawed on ice and washed with NH_4_HCO_3_ buffer twice to remove glycerol. Afterwards, samples were resuspended in 200 μL of NH_4_HCO_3_ buffer (50 mM, pH 7.8) containing 1 mM UDPGA, 1x× NADPH regeneration system and 1 mM propranolol and incubated for the indicated amounts of time at 37 °C and 300 rpm. After incubation, 5 μL of 4-methoxypropranolol (200 µg/mL) were added to each diploid strain reaction sample as an internal standard. Afterwards, the mixtures were extracted thrice with an equal volume of ethyl acetate, and then the organic phases were combined and dried under N_2_. The extracts were redissolved in methanol and analyzed using LC-MS/MS for 4-hydroxypropranolol formation. The aqueous residues were centrifuged at 14,100× *g* for 5 min to obtain supernatants and then analyzed using LC-MS/MS as well for glucuronide detection. In addition, the reaction mixtures from CAD200 samples were centrifuged at 14,100× *g* for 5 min and the supernatants were used directly for LC-MS/MS analysis of propranolol glucuronides.

### 2.7. LC-MS Analysis

The analysis was carried out using an Agilent Technologies 1290 II Infinity high-performance liquid chromatograph coupled with an Agilent 6495 triple quadrupole mass spectrometer (Agilent Technologies, Waldbronn, Germany). Separation of propranolol, propranolol glucuronides, hydroxypropranolols, hydroxypropranolol glucuronides, and 4-methoxypropranolol was performed on an Agilent Poroshell phenyl hexyl column (100 mm × 3.0 mm, 1.9 μm).

For the separation of propranolol, propranolol glucuronides, and hydroxypropranolol glucuronides, the following chromatographic conditions were employed: a flow rate of 0.4 mL/min, an injection volume of 5 μL, and a column temperature of 30 °C. The initial mobile phase consisted of 95% water with 0.1% formic acid and 10 mM ammonium formate (A), and 5% acetonitrile with 0.1% formic acid, 10% water, and 10 mM ammonium formate (B). The proportion of mobile phase A was gradually decreased to 60% over 14 min, and then further reduced to 5% between 14 and 15 min. Subsequently, mobile phase A was maintained at 5% until the end of the run, which occurred at 17 min. 

Similar conditions were applied for separating propranolol, 4-hydroxypropranolol, 5-hydroxypropranolol, 7-hydroxypropranolol and 4-methoxypropranolol; however, an adjusted mobile phase gradient was used: Starting from 95% A, the amount of A was decreased to 60% within 25 min and to 5% from 25 to 26 min. Then, A was maintained at 5% until the end (28 min).

The details of the source parameters and transitions of analytes are listed in Table 3. The presented peak areas were provided by transitions of the highest intensity.

## 3. Results

### 3.1. Optimization of the Biotransformation Protocol

The previously published protocol for CYP-UGT activity assays with diploid fission yeasts was established using proluciferin probe substrates [19]. For monitoring of propranolol metabolism, this protocol needed to be optimized as this is a much more lipophilic substrate with a logD (7.4) value of 1.2 [36].

#### 3.1.1. Liquid–Liquid Extraction with Ethyl Acetate

**Figure 2:**
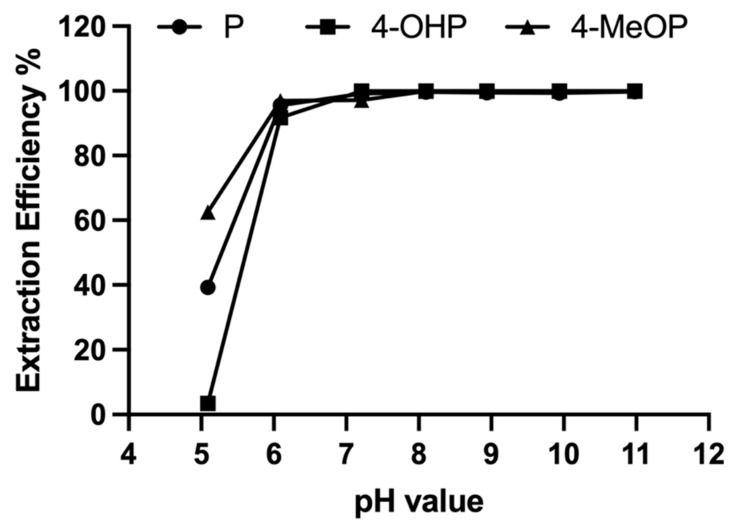
Extraction efficiency of propranolol (P), 4-hydroxypropranolol (4-OHP), and 4-methoxypropranolol (4-MeOP) at different pH values as indicated.

In the proluciferin assays, activity of CYPs and UGTs was directly monitored by measuring luminescence. In the current study, the substrate and its metabolites needed to be extracted from the reaction mixture in order to be subsequently analyzed using LC-MS/MS. Therefore, liquid–liquid extraction was performed using ethyl acetate as previously performed in P450 metabolization studies [18]. An extraction efficiency assay was performed testing different pH levels of the buffer system as this parameter was expected to be a key factor to influence the extraction efficiency. Using a 50 mM ammonium hydrogen carbonate buffer (adjusted to pH 5, 6, 7, 8, 9, 10, or 11) and ethyl acetate as the organic solvent, the three compounds (propranolol, 4-hydroxypropranolol, and 4-methoxypropranolol) were extracted and analyzed. Extraction efficiencies were evaluated as peak areas in the resulting chromatograms. For all three compounds, the extraction efficiency was lower than 65% at pH 5 (Figure 2). From pH 6 to 11, the peak area of extracted analytes was found to be stable and the extraction efficiency was above 95%. The extraction efficiency was calculated as follows:

$$\mathrm{Extraction}\;\mathrm{efficiency}=\frac{{\mathrm{Peak}\;\mathrm{Area}\;}_{\mathrm{extracts}}}{{{\mathrm{Peak}\;\mathrm{Area}}_{\mathrm{extracts}}+\mathrm{Peak}\;\mathrm{Area}\;}_{\mathrm{residues}}}\times 100\%$$

where Peak Area_extracts_ is the peak area of a compound after extraction by ethyl acetate; and Peak Area_residues_ is the peak area of a compound remaining in the aqueous residue after extraction.

Therefore, the reaction buffer used in this study (50 mM ammonium hydrogen carbonate, pH 7.8) was suitable for the extraction. Furthermore, 4-methoxypropranolol was used as an internal standard for the extraction because of its similar extraction trends at different pH values.

#### 3.1.2. Long-Term Storage for Enzyme Bags

In order to reduce batch-to-batch variations in the enzyme bag assays, it is desirable to produce a large number of enzyme bags from a single culture, freeze them, and use them continuously throughout a given project. However, it had to be confirmed that the enzyme activities did not decrease over the time of storage. Therefore, the enzymatic activity during long-term storage was monitored. For this purpose, the production of 4-hydroxypropranolol from propranolol in SAN308 enzyme bag samples was monitored to evaluate the degradation of the activity after deep-freezer storage (−80 °C) for 1 day, 1 week, or 3 months, respectively, and compared with the freshly made ones. All enzyme bags were produced from the same culture and stored in a deep freezer for all biotransformation assays. The activity of the freshly made enzyme bags was defined as 100%. 

**Figure 3:**
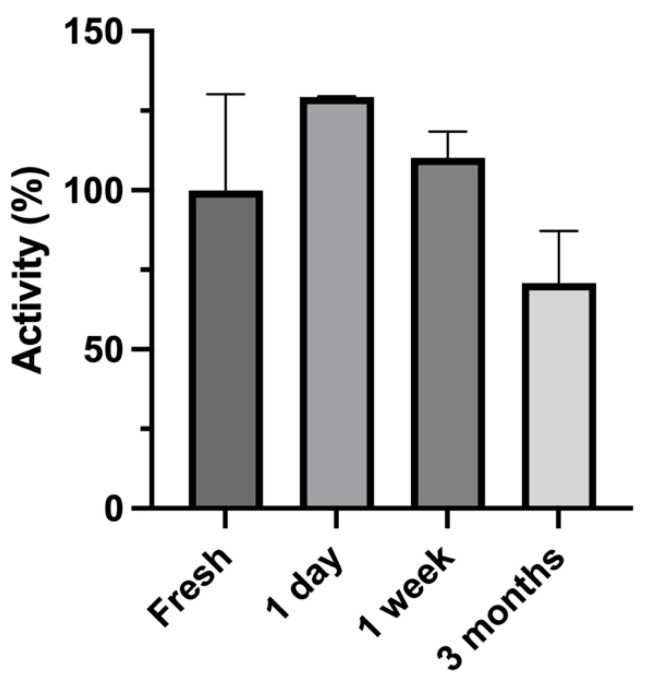
Comparison of the activity of freshly made SAN308 enzyme bags and those stored in a deep freezer (−80 °C) for the times indicated. All reactions were performed in triplicate.

No statistically significant difference in the activities between the freshly made samples and those stored for different time intervals was observed (Figure 3). Thus, stored enzyme bags can be used for at least three months after preparation.

#### 3.1.3. Optimization of the CYP Reaction Time for Diploid Yeast

In our previous studies, different reaction times were used in the enzyme bag method for UGT-catalyzed glucuronidation and P450-catalyzed hydroxylation [30,34]. The diploid yeast used in this study co-expressed both CYP2D6 and one of the four different UGTs (Table 2). Thus, the most suitable reaction time for CYP2D6 was evaluated by monitoring the production of the relative abundances of the metabolite after five different reaction times (2 h, 4 h, 8 h, 16 h, 24 h) using strain SAN308 (which co-expresses CYP2D6 and UGT1A9).

**Figure 4:**
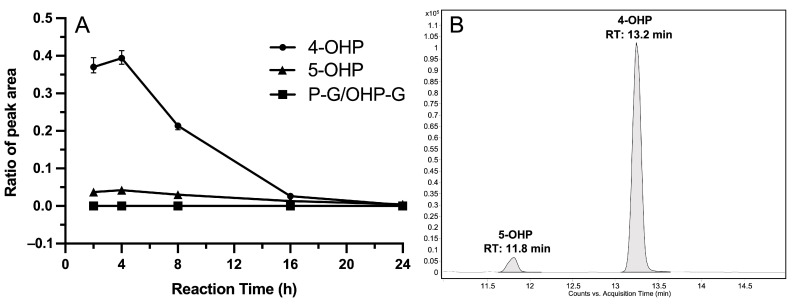
Yield of hydroxypropranolol isomers and glucuronic acid conjugates at different reaction times (**A**) and the chromatogram of hydroxy propranolol isomers (**B**) obtained from SAN308-dependent biotransformation of propranolol. (**A**) Ratio of peak area is the ratio of peak area of 4-/5-OHP divided by peak area of 4-MeOP; P-G, propranolol glucuronides; OHP-G, hydroxy propranolol glucuronides. (**B**) Ion transition *m*/*z* 276→58 was monitored for 4-hydroxypropranolol (4-OHP) and 5-hydroxypropranolol (5-OHP). All reactions were performed in triplicate.

For the production of 4- and 5-hydroxypropranolol, the highest yield was reached at a reaction time of 4 h (Figure 4A). Afterwards, the amount of 4- and 5-hydroxypropranolol decreased to nearly zero as the reaction time increased to 24 h. To cope with potential differences in extraction yields, the production of 4- and 5-hydroxypropranolol was determined by calculating the ratio of the peak area of 4- and 5-hydroxypropranolol to 4-methoxypropranolol. Unexpectedly, no propranolol glucuronides or hydroxy propranolol glucuronides were detected at any reaction time. If they were formed at all, their concentrations were lower than the detection limit. 

Additional experiments were conducted by incubating 4-hydroxypropranolol in NH_4_HCO_3_ buffer for 2, 4, 8, 16, and 24 h. Similar to Figure 4A, the peak area of 4-hydroxypropranolol showed a decreasing trend (data shown in supplementary material), which could be attributed to its chemical instability in vitro. Finally, 4 h was used as the reaction time for producing 4-hydroxypropranolol in SAN308 strain samples.

### 3.2. Influence of Four UGTs on CYP2D6 Activity

**Figure 5:**
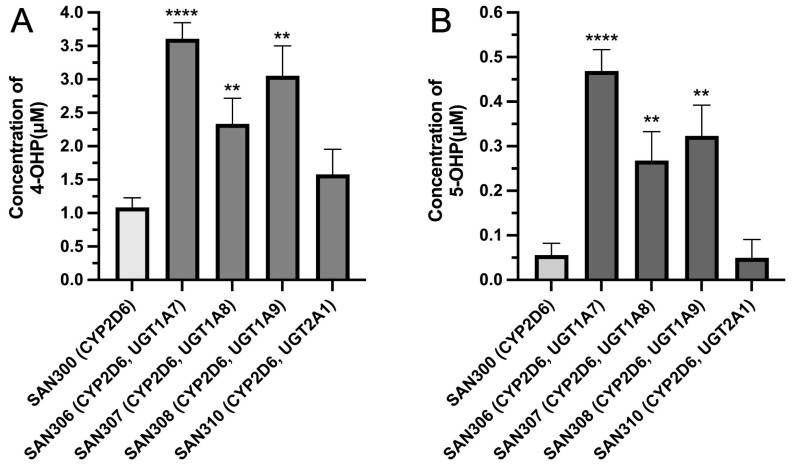
Comparison of the production of 4-hydroxypropranolol (**A**) and 5-hydroxypropranolol (**B**) in CYP2D6-UGT co-expression yeast samples with the control CYP2D6 strain. All reactions were performed in triplicate. All enzyme bags used were stored for <3 months. * *p* < 0.05, ** *p* < 0.01, *** *p* < 0.005, **** *p* < 0.0001.

The optimized enzyme bag method for diploid yeasts was used for the biotransformation of propranolol by four diploid yeast strains which co-express CYP2D6 and one of four UGTs (UGT1A7, UGT1A8, UGT1A9, or UGT2A1). Those four human UGTs were previously shown to catalyze the glucuronidation of propranolol and/or 4-hydroxypropranolol [30]. The diploid yeast strain SAN300 that only expresses CYP2D6 and human CPR was used as a control. Both cofactors (an NADPH regeneration system and UDPGA) were added to the reaction system to enable both the hydroxylation and the glucuronidation reaction. 4-Hydroxypropranolol and 5-hydroxypropranolol were identified via LC-MS/MS comparison with reference standards in all samples (Figure 4B). The concentrations of 4-hydroxypropranolol and 5-hydroxypropranolol were calculated using a standard calibration curve and all the results were normalized using qPCR data of CYP2D6 expression in five diploid strains as reported in the previous study [19]. In comparison with control CYP2D6 samples, the production of 4-hydroxypropranolol using UGT1A7, UGT1A8 and UGT1A9 co-expression was significantly enhanced by a factor of 3.3, 2.1, or 2.8, respectively (Figure 5A). For the production of 5-hydroxypropranolol, the yields increased by 8.4, 4.8, and 5.8 times in UGT1A7, UGT1A8, or UGT1A9 co-expression samples as compared with control CYP2D6 samples (Figure 5B). In contrast, in biotransformation with strain SAN310 (which co-expresses CYP2D6 and UGT2A1), the production of either 4-hydroxypropranolol or 5-hydroxypropranolol showed no significant alteration compared with CYP2D6 control samples.

**Figure 6:**
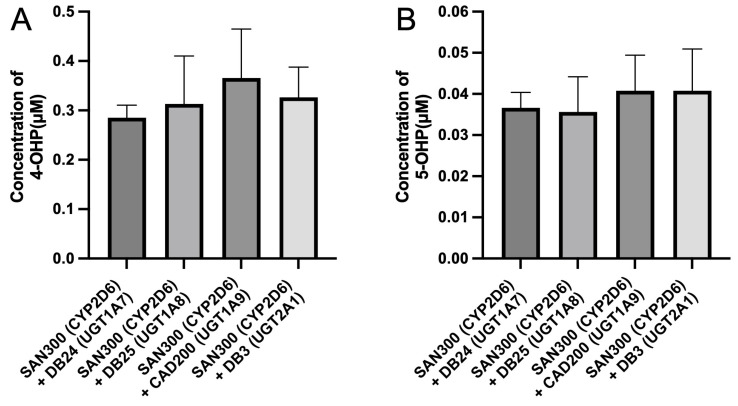
Comparison of the production of 4-hydroxypropranolol (**A**) and 5-hydroxypropranolol (**B**) in mixed samples of control CYP2D6 samples (SAN300) and four UGT single-expression yeast strains (DB24, DB25, CAD200 and DB3). All reactions were performed in triplicate. All enzyme bags used were stored for <3 months.

As a further control experiment, mixtures of enzyme bags prepared from SAN300 (expressing CYP2D6 only) and from one out of four strains (DB24, DB25, CAD200, or DB3) that express single UGTs were also tested in biotransformation experiments. The idea behind this approach was that as the P450 and the UGTs are present in different enzyme bags, there is no possibility for their protein–protein interaction and, thus, they should not influence each other’s activities. In other words, it should not matter which UGT-expressing strain was used in these experiments. As hypothesized, the amounts of both products (4-hydroxypropranolol and 5-hydroxypropranolol) were found not to be significantly different from each other in any of these reactions (Figure 6A,B). This data suggests that the activity differences observed in the CYP-UGT co-expressing strains (Figure 5) are indeed due to interactions between these enzymes.

Furthermore, the aqueous residue obtained from the combined incubations with SAN300 and one of the four UGT strains was found to contain two propranolol glucuronides at similar production rates to those previously reported [30], indicating that the activity of UGTs in single-expression yeast strains remained unaffected even if used in this mixedstrain incubation. No hydroxypropranolol glucuronides were detected in any of the reactions, which was expected as CYP2D6 and UGTs are not physically proximate in this assay, making subsequent glucuronidation after hydroxylation unlikely.

### 3.3. UGT Activities

**Figure 7:**
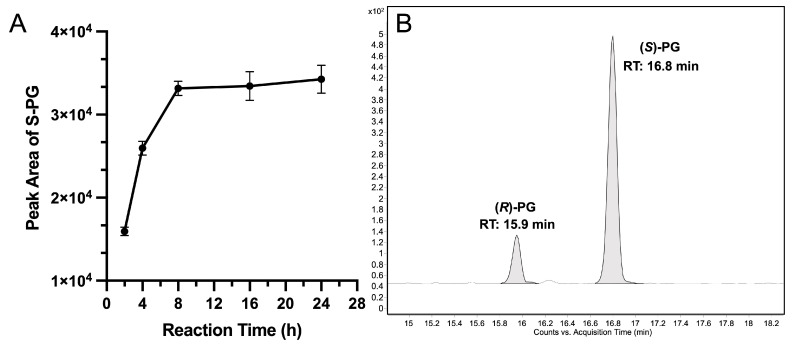
Yield of (*S*)-propranolol glucuronide at different reaction times (**A**) and chromatogram of propranolol glucuronides (**B**) produced by enzyme bags obtained from strain CAD200 (expressing human UGT1A9). Ion transition *m*/*z* 436→116 was monitored for (*R*)-propranolol glucuronide (*R*-PG) and (*S*)-propranolol glucuronide (*S*-PG).

The aqueous residues obtained after ethyl acetate extraction of CYP2D6-UGT-expressing diploid yeast samples were analyzed for glucuronides. Unexpectedly, no UGT activity towards propranolol was found in any of the CYP2D6-UGT co-expression samples (Figure 4A). In contrast, for all four UGTs, activity towards propranolol or 4-hydroxypropanolol was demonstrated before upon recombinant expression in fission yeast [30]. However, in the present study, the reaction conditions were slightly different. As a control for the experimental setup, the biotransformation of racemic propranolol by UGT1A9 over time was, therefore, exemplarily monitored using the haploid yeast strain CAD200, applying the reaction protocol developed for the diploid yeast strain SAN308 described above. For quantitation, the peak area of (*S*)-propranolol glucuronide was used. It was found that product yield reached a maximum at 8 h and then remained stable until 24 h (Figure 7A). The observed activities were in a similar range as reported before [30], with larger peak areas found for (*S*)- than (*R*)-propranolol glucuronide (Figure 7B). 

This data demonstrates that the new protocol developed in this study is well suited to the UGT-dependent biotransformation of propranolol. Thus, the observed lack of propranolol glucuronides found in the diploid strain experiments described above is not due to the reaction conditions but to strongly reduced activities of the UGTs that were co-expressed with CYP2D6.

## 4. Discussion

Although protein–protein interactions between CYPs and UGTs have been studied for decades, their consequences for the activity of the enzymes involved are not well understood yet. Upon CYP-UGT co-expression in a variety of host systems, activity changes had been observed in experiments that involved human CYP1A2, CYP2C9, or CYP3A4, as well as UGT1A1, UGT1A6, UGT1A7, UGT1A9, or UGT2B7, respectively [12,14,15,16]. Next to the CYP3A family, CYP2D6 is arguably one of the most important human drug-metabolizing enzymes. Moreover, the CYP2D6 gene is highly polymorphic, and varying CYP2D6 activities are associated with both adverse drug reactions and reduced drug efficacy [37]. Very recently, we have established a recombinant fission yeast system in which each one of the 19 human members of the UGT families 1 and 2 is co-expressed with either CYP2C9, CYP2D6, or CYP4Z1. A total of 72 interactions between CYPs and UGTs in these new strains were observed using proluciferin probe substrates [19]. With respect to CYP2D6, co-expression of eleven UGTs (including UGT1A7) led to a statistically significant activity decrease, while six (including UGT1A8, UGT1A9, and UGT2A1) caused an increase and the remaining two had no effect. 

It was the aim of the present study to expand these studies using the drug propranolol, which is a beta-receptor blocker used in the treatment of various cardiovascular disorders [22]. This compound was chosen because it is metabolized by both CYP2D6 and a number of UGTs. More specifically, CYP2D6 has been found to be responsible for the hydroxylation of propranolol, thereby producing the phase I metabolites 4-, 5-, and 7-hydroxypropranolol [31,38,39,40]. We have recently demonstrated that propranolol can be glucuronidated by UGT1A7, UGT1A9, UGT1A10, and UGT2A1, while its CYP2D6 metabolite 4-hydroxypropranolol is a substrate for UGT1A7, UGT1A8, UGT1A9, and UGT2A1 [30]. Therefore, diploid yeast strains that co-express CYP2D6 and each of the latter four UGTs were used in this study for investigating their mutual influences.

The biotransformation method was adapted and optimized for the diploid yeast system in this study. In our previously published enzyme bag method [18], recombinant fission yeast cells are permeabilized by detergent, forming holes on the cell membrane and thus facilitating small molecules (such as substrate and cofactors) and hydrophilic products to get in and out of the cells. However, lipophilic substrates (and their metabolites) are likely to be embedded in the cellular membranes of the enzyme bags. In such cases, a liquid–liquid extraction of the metabolites from the aqueous reaction buffer is required. In one of our previous studies, ethyl acetate was used for the extraction of CYP metabolites of testosterone [18]. In this study, the extraction efficiency of ethyl acetate on propranolol, 4-hydroxypropranolol, and 4-methoxypropranolol at different pH values (ranging from pH 5 to 11) was investigated to find the best condition. As shown in Figure 2, the extraction of all three compounds is not effective when the pH value is below 6. As the pH value increased, the extracted amount of all three compounds tended to be higher and stable according to the peak area in the respective chromatograms. Therefore, the reaction buffer used in this study (50 mM ammonium hydrogen carbonate, pH 7.8) was compatible with the extraction procedure. Thus, ethyl acetate was used for the extraction of hydroxypropranolols for all biotransformation samples and 4-methoxypropranolol was used as the internal standard. In contrast, the aqueous residue was analyzed for glucuronides since they are highly hydrophilic and not easily extracted by ethyl acetate.

In any biological reaction system, batch-to-batch variability is a concern. One solution to this problem is to use cells from a single batch throughout a given project. However, this requires a method for preservation of the biocatalysts. In one of our previous studies, we mentioned the possibility of long-term storage for enzyme bag samples, but the question of activity alteration during long-term storage was not addressed [34]. Therefore, in this study, we monitored the conversion of propranolol to 4-hydroxypropranolol in SAN308 enzyme bags (which contained CYP2D6 and UGT1A9) that were initially frozen and stored for different time spans. It was found that there were no statistically significant differences in the activities between the freshly made aliquots and those stored for up to three months. This allowed us to perform all biotransformations from a single batch of enzyme bags prepared from each strain used in this study. 

Both hydroxylation and glucuronidation reactions were investigated in the biotransformation with CYP-UGT co-expression yeast strains here. In our previous studies with enzyme bags, optimal reaction times varied for CYPs and UGTs [18,30]. Several different reaction times were, therefore, tested in this study to find the most suitable one for the potential enzymatic chain reaction. As shown in Figure 4A, the production of 4-hydroxypropranolol reached the highest yield at the reaction time of 4 h. Afterwards, the amount of 4-hydroxypropranolol decreased as the reaction time increased. Two major hydroxy propranolol products were found in all CYP2D6-UGT co-expression samples. These were identified as 4-hydroxypropranolol and 5-hydroxypropranolol via LC-MS/MS comparison with reference standards. Formation of *N*-desisopropylated metabolites [26] was also observed at lower levels but no increased production was found as the incubation time increased, which means the generation of *N*-desisopropylated metabolites may not be the reason for the decrease in 4-hydroxypropranolol. Further experiments were carried out by incubating 4-hydroxypropranolol in NH_4_HCO_3_ buffer for the same time intervals as in SAN308 reactions. The results revealed a decline in the peak area of 4-hydroxypropranolol, similar to that shown in Figure 4A. Details are provided as Supplementary Figure S1. This finding suggests that the degradation of 4-hydroxypropranolol does not reach saturation within 4 h and follows a first-order kinetic behavior. This outcome is in line with previous investigations that reported the instability of 4-hydroxypropranolol in aqueous solution and recommended that urine samples containing this compound be analyzed promptly after collection [41,42,43]. Therefore, the reaction time of 4 h was chosen for the biotransformation of the CYP2D6-UGT co-expression yeast as the degradation of 4-hydroxypropranolol had not yet caused a significant effect and no glucuronides were found or they were lower than the detection limit at any reaction time. 

Under these optimized conditions, the influence of UGTs on CYP2D6 activity was investigated by monitoring the production of 4- and 5-hydroxypropranolol. In our previous study [19], the activity of CYP2D6 towards a proluciferin probe substrate was significantly increased upon co-expression of six UGTs (UGT1A4, 1A9, 1A10, 2A1, 2A3, and 2B10). On the other hand, co-expression of eleven UGTs (UGT1A3, 1A5, 1A6, 1A7, 1A8, 2A2, 2B4, 2B7, 2B15, 2B17, and 2B28) led to activity decreases, while co-expression of the remaining two UGTs (UGT1A1 and 2B11) had no statistically significant effect. The increases in CYP2D6 activities varied between a factor of 1.4 and 4.3 and the reduction was between 1.3-fold and 3.6-fold. In the present study, UGT1A7, UGT1A8, and UGT1A9 increased the yield of 4-hydroxypropranolol by 3.3-, 2.1-, and 2.8-fold compared with control CYP2D6 samples. Similarly, co-expression of UGT1A7, UGT1A8, and UGT1A9 increased the production of 5-hydroxypropranolol to a factor of 8.4, 4.8, and 5.8. The observed positive effect of UGT1A9 on CYP2D6 activity in propranolol hydroxylation is consistent with the increased activity (around 1.8-fold) of CYP2D6 with UGT1A9 co-expression on the metabolism of the proluciferin probe substrate [19]. In contrast to the incubation of the proluciferin as substrates, UGT2A1 showed no significant influence on the CYP2D6-dependent hydroxylation of propranolol. UGT1A7, UGT1A8, and UGT2A1 showed different tendencies for CYP2D6 activities compared with our previous results, indicating that such interactions also depend on the substrate under study. Based on the expression data obtained from the Human Protein Atlas database (https://www.proteinatlas.org/, accessed on 25 May 2023), CYP2D6 is co-expressed with UGT1A7, UGT1A8, UGT1A9, and UGT2A1 in many human tissues with various expression levels. CYP2D6 is strongly co-expressed with UGT1A9 in the liver and with UGT1A8 in the small intestine. Co-expression of CYP2D6 and UGT1A7 is observed in the colon, cerebellum, and choroid plexus. Additionally, the cerebellum and choroid plexus also exhibit co-expression of CYP2D6 and UGT2A1, with exclusive co-expression of these enzymes in the pituitary gland. These findings suggest that the observed influence of UGT on CYP2D6 in in vitro experiments may have relevance for the tissues where they are co-expressed, potentially affecting the metabolism of propranolol in those specific locations.

As an additional control, mixtures of enzyme bags that either expressed CYP2D6 or an UGT were also tested in biotransformation experiments. As there was no possibility of a CYP–UGT interaction in this experimental setup, the different combinations were expected to give similar results. Indeed, this was the outcome of the CYP2D6 activity experiments. Moreover, two propranolol glucuronic diastereomers were found in all mixed groups with production rates at a similar level as described before [30]. This data demonstrates that the UGTs employed in this study were active under the reaction conditions used as long as they were not co-expressed with CYP2D6.

As a further control of the results obtained in the present study, a haploid fission yeast strain which solely expresses UGT1A9 (CAD200) was tested for its activity towards racemic propranolol at different reaction times, using the same method as described above. Both (*R*)- and (*S*)-propranolol glucuronide were found as expected, with production of (*S*)-propranolol glucuronide reaching the maximum yield at 8 h. These results again demonstrate that the experimental setup was suitable for monitoring UGT activities. If the CYP2D6-UGT co-expressing enzyme bags had produced any propranolol or hydroxypropranolol glucuronides, they would have been detectable; alternatively, their concentrations were lower than the detection limit if they were formed at all. 

In our previous study, the effect of CYP co-expression (using CYP2C9, CYP2D6, and CYP4Z1) on the activities of five UGTs (UGT1A4, 1A9, 2A3, 2B7, and 2B28) was monitored [19]. The activities of UGT1A1, UGT1A9, and UGT2B28 were found to be reduced by all three CYPs, while the activity of UGT2B7 was not influenced at all. Co-expression of CYP4Z1 suppressed the activity of UGT2A3 while no effect was found in the CYP2C9 and CYP2D6 co-expressing strains. Overall, neither in our previous study nor in the present work was a positive influence of any CYP on the activity of any UGTs ever observed. If there was an influence, it was always detrimental.

Multiple studies have shown that the polymorphisms of UGTs may influence the enzymatic activity towards different substrates. For example, UGT1A6*2 has been found to metabolize 3-*O*-methyl-dopa and methyl-salicylate at 41–74% of the wild-type level, while the metabolism of 1-naphthol, 3-iodopenol, 7-hydroxycoumarin, and 7-hydroxy-4-methylcoumarin remains normal [44]. Additionally, serotonin, 5-hydroxytryptophol, 4-nitrophenol, acetaminophen, and valproic acid were found to have two-fold higher glucuronidation by UGT1A6*2 [45]. Another example is UGT1A9*3, which shows dramatically decreased glucuronidation activity towards SN-38 with only 3.8% of the wild-type level [46]. However, −275 and −2152 SNPs of UGT1A9 showed 2.2- to 2.3-fold higher glucuronidation activity towards propofol and 1.9- to 2.1-fold elevated activity on mycophenolic acid glucuronidation [47]. In this study, we discovered that co-expression of CYPs may influence the activity of the UGTs in a way that can be compared to the polymorphism. The conformation of the UGT and the substrate, or their position in the membrane, might be changed due to interactions with CYPs. The similarity is that in both cases, activities of the UGTs are changed depending on the substrate. The negative effects on UGTs observed in this study are the strongest we have ever seen in any UGT-catalyzed reaction. Therefore, when studying an enzymatic chain reaction in co-expression microbe systems, this kind of serious suppression should be taken into account. 

Additionally, it is worth noting that propranolol glucuronides and 4-hydroxypropranolol glucuronides have been previously detected in human urine [39,48,49], indicating the complexity of CYP-UGT interactions in vivo due to the involvement of multiple CYP and UGT isoforms. As such, the suppressed activity of UGTs towards propranolol and hydroxypropranolol glucuronidation observed in our study might be restored or increased by other CYP isoforms, as seen with the increased activity of UGT2B7 on morphine glucuronidation by CYP3A4 [12]. Therefore, our enzyme bag method with diploid CYP–UGT co-expression yeast strains serves as an ideal model for investigating individual protein–protein interactions without interferences from other isoforms.

## 5. Conclusions

In this study, we successfully optimized the enzyme bag method for diploid yeast incubation and demonstrated the feasibility of long-term storage of enzyme bags. Using the optimized method and stored enzyme bags, the mutual interaction between CYP2D6 and four UGTs was investigated. Our results showed that UGT1A7, UGT1A8 and UGT1A9 had a significant enhancing effect on the activity of CYP2D6, while UGT2A1 had no influence. On the other hand, the glucuronidation by four UGTs was seriously suppressed by CYP2D6. The use of propranolol as a substrate, which is a widely prescribed drug, provides significant practical implications for the study of CYP and UGT interactions in drug metabolism. In addition, the functional interaction between CYP2D6 and UGT1A subfamilies was observed in this study for the first time. It may be speculated that the reason why UGT2A1 did not show similar positive effects on CYP2D6 as the other UGT1A enzymes was due to differences in the sequence, which may result in the distinct interaction with CYP2D6. In future studies, CYP2D6 co-expression with targeted point mutation of UGTs could be taken into consideration for identifying specific sequences involved in the mutual interaction using biological experiments or computational aids.

## Figures and Tables

**Table 1:** List of primers.

Gene	Forward Primer	Reverse Primer	Product Size
*act1*	5′-GTTATGTCTGGTGGTACCACT-3′	5′-GATCCACCAATCCAGACAGA-3′	140 bp
*CYP2D6*	5′-AGGTCCATTGCCACTTCCAG-3′	5′-CCGAACAGCTGCTAGACCAT-3′	160 bp

**Table 2:** Fission yeast strains used in this study.

Strain	Expressed Protein	Genotype	Reference
DB24	UGT1A7	h- ura4-D18 leu1::pCAD1UGT1A7	[17]
DB25	UGT1A8	h- ura4-D18 leu1::pCAD1UGT1A8	[17]
CAD200	UGT1A9	h- ura4-D18 leu1::pCAD1UGT1A9	[17]
DB3	UGT2A1	h- ura4-D18 leu1::pCAD1UGT2A1	[17]
SAN300	hCPR, CYP2D6	h+/h− ade6-M210/ade6-M216 ura4-D18/ura4-D18 his3.Δ1/his3.Δ1 leu1::pCAD1-CPR/leu1::pCAD1/pREP1-CYP2D6	[19]
SAN306	hCPR, CYP2D6, UGT1A7	h+/h− ade6-M210/ade6-M216 ura4-D18/ura4-D18 his3.Δ1/his3.Δ1 leu1::pCAD1-CPR/leu1::pCAD1-UGT1A7/pREP1-CYP2D6	[19]
SAN307	hCPR, CYP2D6, UGT1A8	h+/h− ade6-M210/ade6-M216 ura4-D18/ura4-D18 his3.Δ1/his3.Δ1 leu1::pCAD1-CPR/leu1::pCAD1-UGT1A8/pREP1-CYP2D6	[19]
SAN308	hCPR, CYP2D6, UGT1A9	h+/h− ade6-M210/ade6-M216 ura4-D18/ura4-D18 his3.Δ1/his3.Δ1 leu1::pCAD1-CPR/leu1::pCAD1-UGT1A9/pREP1-CYP2D6	[19]
SAN310	hCPR, CYP2D6, UGT2A1	h+/h− ade6-M210/ade6-M216 ura4-D18/ura4-D18 his3.Δ1/his3.Δ1 leu1::pCAD1-CPR/leu1::pCAD1-UGT2A1/pREP1-CYP2D6	[19]

**Table 3:** Transitions for all analytes in MRM.

Analytes	Precursor Ions (*m*/*z*)	Product Ions (*m*/*z*)	CE (V)	ESI
Propranolol glucuronide	436	258	12	+
	436	183	16	+
	436	116	28	+
4-/5-Hydroxypropranolol glucuronide	452	276	12	+
	452	116	28	+
	452	72	44	+
4-/5-Hydroxypropranolol	276	116	12	+
	276	72	16	+
	276	58	44	+
4-Methoxypropranolol	290	187	12	+
	290	116	16	+
	290	72	44	+

CE, collision energy; ESI, electrospray ionization; gas temperature, 160 °C; gas flow, 11 L/min; nebulizer, 30 psi; sheath gas heater, 375 °C; sheath gas flow, 12 L/min; capillary, 3000 V; nozzle voltage, 1500 V.

## Data Availability

Raw data are stored with the authors.

## Supplementary Materials

The following supporting information can be downloaded at: https://www.mdpi.com/article/10.3390/cimb45090451/s1. Figure S1: Degradation assay of 4-hydroxypropranolol (4-OHP). The samples were treated under the same conditions as in enzyme bags reactions but without yeast cells. The amount of 4-hydroxypropranolol left at six time points (0 h, 2 h, 4 h, 8 h, 16 h and 24 h) were analyzed and normalized to values at T0 = 0 h. All reactions were done in triplicates.

## References

[1] Meyer U.A. (1996). Overview of enzymes of drug metabolism. J. Pharmacokinet. Biopharm..

[2] Josephy P.D., Guengerich F.P., Miners J.O. (2005). “Phase I and Phase II” drug metabolism: Terminology that we should phase out?. Drug Metab. Rev..

[3] Guengerich F.P. (2017). Intersection of the Roles of Cytochrome P450 Enzymes with Xenobiotic and Endogenous Substrates: Relevance to Toxicity and Drug Interactions. Chem. Res. Toxicol..

[4] Hannemann F., Bichet A., Ewen K.M., Bernhardt R. (2007). Cytochrome P450 systems-biological variations of electron transport chains. Biochim. Biophys. Acta.

[5] Di L., Kerns E.H. (2016). Drug-Like Properties: Concepts, Structure Design and Methods from ADME to Toxicity Optimization.

[6] Rowland A., Miners J.O., Mackenzie P.I. (2013). The UDP-glucuronosyltransferases: Their role in drug metabolism and detoxification. Int. J. Biochem. Cell Biol..

[7] Guillemette C., Levesque E., Rouleau M. (2014). Pharmacogenomics of human uridine diphospho-glucuronosyltransferases and clinical implications. Clin. Pharmacol. Ther..

[8] Miners J.O., Rowland A., Novak J.J., Lapham K., Goosen T.C. (2021). Evidence-based strategies for the characterisation of human drug and chemical glucuronidation in vitro and UDP-glucuronosyltransferase reaction phenotyping. Pharmacol. Ther..

[9] Taura K.I., Yamada H., Hagino Y., Ishii Y., Mori M.A., Oguri K. (2000). Interaction between cytochrome P450 and other drug-metabolizing enzymes: Evidence for an association of CYP1A1 with microsomal epoxide hydrolase and UDP-glucuronosyltransferase. Biochem. Biophys. Res. Commun..

[10] Taura K., Naito E., Ishii Y., Mori M.A., Oguri K., Yamada H. (2004). Cytochrome P450 1A1 (CYP1A1) inhibitor alpha-naphthoflavone interferes with UDP-glucuronosyltransferase (UGT) activity in intact but not in permeabilized hepatic microsomes from 3-methylcholanthrene-treated rats: Possible involvement of UGT-P450 interactions. Biol. Pharm. Bull..

[11] Fremont J.J., Wang R.W., King C.D. (2005). Coimmunoprecipitation of UDP-glucuronosyltransferase isoforms and cytochrome P450 3A4. Mol. Pharmacol..

[12] Takeda S., Ishii Y., Iwanaga M., Mackenzie P.I., Nagata K., Yamazoe Y., Oguri K., Yamada H. (2005). Modulation of UDP-glucuronosyltransferase function by cytochrome P450: Evidence for the alteration of UGT2B7-catalyzed glucuronidation of morphine by CYP3A4. Mol. Pharmacol..

[13] Takeda S., Ishii Y., Iwanaga M., Nurrochmad A., Ito Y., Mackenzie P.I., Nagata K., Yamazoe Y., Oguri K., Yamada H. (2009). Interaction of cytochrome P450 3A4 and UDP-glucuronosyltransferase 2B7: Evidence for protein-protein association and possible involvement of CYP3A4 J-helix in the interaction. Mol. Pharmacol..

[14] Ishii Y., Koba H., Kinoshita K., Oizaki T., Iwamoto Y., Takeda S., Miyauchi Y., Nishimura Y., Egoshi N., Taura F. (2014). Alteration of the function of the UDP-glucuronosyltransferase 1A subfamily by cytochrome P450 3A4: Different susceptibility for UGT isoforms and UGT1A1/7 variants. Drug Metab. Dispos..

[15] Miyauchi Y., Nagata K., Yamazoe Y., Mackenzie P.I., Yamada H., Ishii Y. (2015). Suppression of Cytochrome P450 3A4 Function by UDP-Glucuronosyltransferase 2B7 through a Protein-Protein Interaction: Cooperative Roles of the Cytosolic Carboxyl-Terminal Domain and the Luminal Anchoring Region. Mol. Pharmacol..

[16] Miyauchi Y., Tanaka Y., Nagata K., Yamazoe Y., Mackenzie P.I., Yamada H., Ishii Y. (2020). UDP-Glucuronosyltransferase (UGT)-mediated attenuations of cytochrome P450 3A4 activity: UGT isoform-dependent mechanism of suppression. Br. J. Pharmacol..

[17] Dragan C.A., Buchheit D., Bischoff D., Ebner T., Bureik M. (2010). Glucuronide Production by Whole-Cell Biotransformation Using Genetically Engineered Fission Yeast Schizosaccharomyces pombe. Drug Metab. Dispos..

[18] Yan Q., Machalz D., Zollner A., Sorensen E.J., Wolber G., Bureik M. (2017). Efficient substrate screening and inhibitor testing of human CYP4Z1 using permeabilized recombinant fission yeast. Biochem. Pharmacol..

[19] Sharma S.S., Sharma S., Zhao J., Bureik M. (2023). Mutual Influence of Human Cytochrome P450 Enzymes and UDP-Glucuronosyltransferases on Their Respective Activities in Recombinant Fission Yeast. Biomedicines.

[20] Bureik M., Schiffler B., Hiraoka Y., Vogel F., Bernhardt R. (2002). Functional Expression of Human Mitochondrial CYP11B2 in Fission Yeast and Identification of a New Internal Electron Transfer Protein, etp1. Biochemistry.

[21] Ikushiro S.-i., Sahara M., Emi Y., Yabusaki Y., Iyanagi T. (2004). Functional co-expression of xenobiotic metabolizing enzymes, rat cytochrome P450 1A1 and UDP-glucuronosyltransferase 1A6, in yeast microsomes. Biochim. Et. Biophys. Acta (BBA)-Gen. Subj..

[22] Routledge P.A., Shand D.G. (1979). Clinical pharmacokinetics of propranolol. Clin. Pharmacokinet..

[23] Brosen K. (1995). Drug interactions and the cytochrome P450 system. The role of cytochrome P450 1A2. Clin. Pharmacokinet..

[24] Yoshimoto K., Echizen H., Chiba K., Tani M., Ishizaki T. (1995). Identification of human CYP isoforms involved in the metabolism of propranolol enantiomers--N-desisopropylation is mediated mainly by CYP1A2. Br. J. Clin. Pharmacol..

[25] Zhou Q., Yao T.W., Zeng S. (2002). Chiral reversed phase high-performance liquid chromatography for determining propranolol enantiomers in transgenic Chinese hamster CHL cell lines expressing human cytochrome P450. J. Biochem. Biophys. Methods.

[26] Bichara N., Ching M.S., Blake C.L., Ghabrial H., Smallwood R.A. (1996). Propranolol hydroxylation and N-desisopropylation by cytochrome P4502D6: Studies using the yeast-expressed enzyme and NADPH/O2 and cumene hydroperoxide-supported reactions. Drug Metab. Dispos..

[27] Masubuchi Y., Hosokawa S., Horie T., Suzuki T., Ohmori S., Kitada M., Narimatsu S. (1994). Cytochrome P450 isozymes involved in propranolol metabolism in human liver microsomes. The role of CYP2D6 as ring-hydroxylase and CYP1A2 as N-desisopropylase. Drug Metab. Dispos..

[28] Thompson J.A., Hull J.E., Norris K.J. (1981). Glucuronidation of propranolol and 4′-hydroxypropranolol. Substrate specificity and stereoselectivity of rat liver microsomal glucuronyltransferases. Drug Metab. Dispos..

[29] Johnson J.A., Herring V.L., Wolfe M.S., Relling M.V. (2000). CYP1A2 and CYP2D6 4-Hydroxylate Propranolol and Both Reactions Exhibit Racial Differences. J. Pharmacol. Exp. Ther..

[30] Yang F., Liu S., Wolber G., Bureik M., Parr M.K. (2022). Complete Reaction Phenotyping of Propranolol and 4-Hydroxypropranolol with the 19 Enzymes of the Human UGT1 and UGT2 Families. Int. J. Mol. Sci..

[31] Harps L.C., Schipperges S., Bredendiek F., Wuest B., Borowiak A., Parr M.K. (2020). Two dimensional chromatography mass spectrometry: Quantitation of chiral shifts in metabolism of propranolol in bioanalysis. J. Chromatogr. A.

[32] Oatis Jr J.E., Russell M.P., Knapp D.R., Walle T. (1981). Ring-Hydroxylated Propranolol: Synthesis and beta-Receptor Antagonist and Vasodilating Activities of the Seven Isomers. J. Med. Chem..

[33] Livak K.J., Schmittgen T.D. (2001). Analysis of relative gene expression data using real-time quantitative PCR and the 2(T)(-Delta Delta C) method. Methods.

[34] Sharma S., Durairaj P., Bureik M. (2020). Rapid and convenient biotransformation procedure for human drug metabolizing enzymes using permeabilized fission yeast cells. Anal. Biochem..

[35] Alfa C., Fantes P., Hyams J., McLeod M., Warbrick E. (1993). Experiments with Fission Yeast. A Laboratory Course Manual.

[36] Yamashita T., Nishimura I., Nakamura T., Fukami T. (2009). A system for LogD screening of new drug candidates using a water-plug injection method and automated liquid handler. JALA J. Assoc. Lab. Autom..

[37] Taylor C., Crosby I., Yip V., Maguire P., Pirmohamed M., Turner R.M. (2020). A Review of the Important Role of CYP2D6 in Pharmacogenomics. Genes..

[38] Walle T., Oatis J.E., Walle U.K., Knapp D.R. (1982). New ring-hydroxylated metabolites of propranolol: Species differences and stereospecific 7-hydroxylation. Drug Metab. Dispos..

[39] Walle T., Walle U.K., Olanoff L.S. (1985). Quantitative account of propranolol metabolism in urine of normal man. Drug Metab. Dispos. Biol. Fate Chem..

[40] Distlerath L.M., Reilly P.E., Martin M.V., Davis G.G., Wilkinson G.R., Guengerich F.P. (1985). Purification and characterization of the human liver cytochromes P-450 involved in debrisoquine 4-hydroxylation and phenacetin O-deethylation, two prototypes for genetic polymorphism in oxidative drug metabolism. J. Biol. Chem..

[41] Herring V.L., Johnson J.A. (1993). Direct high-performance liquid chromatographic determination in urine of the enantiomers of propranolol and its major basic metabolite 4-hydroxypropranolol. J. Chromatogr. B Biomed. Sci. Appl..

[42] Fitzgerald J., O’DONNELL S.R. (1971). Pharmacology of 4-hydroxypropranolol, a metabolite of propranolol. Br. J. Pharmacol..

[43] Pritchard J.F., Schneck D.W., Hayes Jr A.H. (1979). Determination of propranolol and six metabolites in human urine by high-pressure liquid chromatography. J. Chromatogr. B Biomed. Sci. Appl..

[44] Ciotti M., Marrone A., Potter C., Owens I.S. (1997). Genetic polymorphism in the human UGT1A6 (planar phenol) UDP-glucuronosyltransferase: Pharmacological implications. Pharmacogenetics.

[45] Krishnaswamy S., Hao Q., Al-Rohaimi A., Hesse L.M., von Moltke L.L., Greenblatt D.J., Court M.H. (2005). UDP glucuronosyltransferase (UGT) 1A6 pharmacogenetics: II. Functional impact of the three most common nonsynonymous UGT1A6 polymorphisms (S7A, T181A, and R184S). J. Pharmacol. Exp. Ther..

[46] Villeneuve L., Girard H., Fortier L.C., Gagne J.F., Guillemette C. (2003). Novel functional polymorphisms in the UGT1A7 and UGT1A9 glucuronidating enzymes in Caucasian and African-American subjects and their impact on the metabolism of 7-ethyl-10-hydroxycamptothecin and flavopiridol anticancer drugs. J. Pharmacol. Exp. Ther..

[47] Girard H., Court M.H., Bernard O., Fortier L.C., Villeneuve L., Hao Q., Greenblatt D.J., von Moltke L.L., Perussed L., Guillemette C. (2004). Identification of common polymorphisms in the promoter of the UGT1A9 gene: Evidence that UGT1A9 protein and activity levels are strongly genetically controlled in the liver. Pharmacogenetics.

[48] Bai S.A., Walle T. (1984). Isolation, purification, and structure identification of glucuronic acid conjugates of propranolol and alprenolol and their ring-hydroxylated metabolites. Drug Metab. Dispos..

[49] Walle T., Conradi E.C., Walle U.K., Fagan T.C., Gaffney T.E. (1980). 4-Hydroxypropranolol and its glucuronide after single and long-term doses of propranolol. Clin. Pharmacol. Ther..

