# Supplementary material for: Mutual Modulation of the Activities of Human CYP2D6 and Four UGTs during the Metabolism of Propranolol
Source: Curr Issues Mol Biol. 2023 Aug 26;45(9):7130–46. doi: 10.3390/cimb45090451 (PMC10527876; doi:10.3390/cimb45090451)
Supplement: Supplementary file 1 [file cimb-45-00451-s001.zip › cimb-2571116-supplementary.pdf]

**Supplementary Figure S1.**

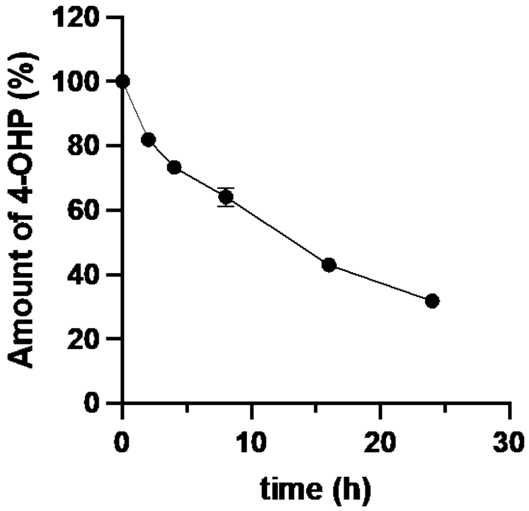


Degradation assay of 4-hydroxypropranolol (4-OHP). The samples were treated under the same conditions as in enzyme bags reactions but without yeast cells. The amount of 4-hydroxypropranolol left at six time points (0 h, 2 h, 4 h, 8 h, 16 h and 24 h) were analyzed and normalized to values at $T_0$ = 0 h. All reactions were done in triplicates.
